# Dynamics of multiple sustainable agricultural intensification practices adoption: Application of the intertemporal multivariate probit model

**DOI:** 10.1371/journal.pone.0314172

**Published:** 2025-02-07

**Authors:** Ali Mohammed Oumer, Michael Burton, Menale Kassie

**Affiliations:** 1 Agricultural and Resource Economics, UWA School of Agriculture and Environment, University of Western Australia, Crawley, WA, Perth, Australia; 2 Ethiopian Institute of Agricultural Research (EIAR), Addis Ababa, Ethiopia; 3 International Centre for Agricultural Research in the Dry Areas (ICARDA), Ariana, Tunis, Tunisia; 4 International Centre of Insect Physiology and Ecology (ICIPE), Nairobi, Kenya; Bangladesh Agricultural University, BANGLADESH

## Abstract

Applying an intertemporal multivariate probit model, we reveal complex complementarity and substitution effects as well as new insights on the drivers of adopting input-intensive and natural resource management (NRM) practices in rural Ethiopia. First, the latent factor that drives each practice is positively and significantly correlated across time, suggesting persistency in adoption decisions. Second, the significant synergies and tradeoffs between the input-intensive and NRM practices underscore that these practices are highly compatible and, hence the importance of promoting technology packages. Third, the covariates that drive adoption significantly differ between practices, reflecting the heterogeneity in farmer behavior. Farm size was associated with the adoption of several input-intensive and NRM practices while off-farm income has the reverse effect. These findings have significant implications for food security policy in sub-Saharan Africa.

## 1. Introduction

Sustainable agricultural intensification (SAI) is seen as a feasible strategy to enhance farm productivity, with a minimal environmental footprint [1–4]. SAI practices have been widely promoted to raise crop yields and food security while also conserving the natural resource base. The premise is that these twin goals can be achieved by fostering synergistic and tradeoff relationships between the practices, conserving nutrients and increasing the productivity of farmers [1–7].

Broadly, there are two types of SAI practices: input-intensive and natural resource management (NRM) practices. The input-intensive practices include external inputs like improved seed (fresh hybrid and open-pollinated varieties recycled at most for three production seasons) and chemical fertilizer. The NRM practices include low-external-input agronomic techniques like as soil and water conservation (SWC) measures, crop residue retention, legume rotation, legume intercropping, reduced tillage, and use of organic manure. These two types of SAI practices are often perceived as incompatible [8–10]. Some argue that input-intensive practices are most appropriate, with a substantial role of the private sector in technology generation and promotion [11–13]. Others advocate the significant role of NRM practices to curb soil degradation and climate variability [14–16]. However, the adoption rates of NRM practices are disappointing despite substantial promotion efforts particularly in sub-Saharan Africa (SSA). In this study, ‘adoption’ is defined in general terms as the use of SAI practices on a farm in a particular year. Most previous adoption studies [17–23] have analyzed the drivers of adoption of individual practices. However, these studies ignore the fact that SAI practices are interdependent [24] and can be driven by complex factors that may relate to tradeoffs and synergistic effects between the practices. A few recent studies have addressed these shortcomings by modelling the adoption of multiple SAI practices and a set of covariates simultaneously in a static framework [8, 9, 25, 26].

Smallholder farmers can make complex sequential adoption decisions due to technological and institutional hurdles in the production process [27, 28]. That is, adopting one technology may drive the adoption of another technology or the future use of the same technology. This outcome could have a positive or negative effect on the subsequent adoption behavior of farmers. In particular, the temporal nature of the adoption of SAI practices is critical but has rarely been investigated while accounting for correlation among the various practices. The likelihood of using SAI practices can depend on whether farmers have tried those practices before and evaluated for accrued benefits against costs. Previous research has considered learning by experience and duration models to investigate the temporal drivers and dynamics of agricultural technologies [29–35]. However, this strand of literature has not captured the intertemporal correlation among the interdependent SAI practices.

This study addresses these shortcomings by investigating the drivers of adoption and intertemporal correlation among the practices. We use a balanced two-wave panel data of 2031 maize producing farm households in Ethiopia. In this paper, balanced panel data is required to investigate the intertemporal adoption process. About 9% of the sample were dropped during balancing because they did not grow maize in both periods. We consider eight SAI practices, consisting of input-intensive and NRM practices adopted in 2009/2010 and 2012/2013. The input-intensive techniques include the use of chemical fertilizer and improved maize seed. The NRM practices involve different low–external input strategies that are mostly implemented to curb soil erosion and reverse land degradation. The selected NRM practices include the use of crop residues, legume rotation, legume intercropping, minimum tillage, soil, and water conservation (SWC) measures, and organic manure.

We contribute to the technology adoption literature by estimating a dynamic multivariate probit (MVP) model that accounts for farmers’ simultaneous adoption decisions and interactions between adoption decisions over time. The dynamic MVP simultaneously models the relationships among multiple practices and a set of covariates by allowing individual-specific unobserved effects to be correlated between the practices both within a period and across periods. Furthermore, we contribute to this literature by modeling adoption drivers or covariates of SAI practices in the intertemporal MVP adoption model which can inform development policy.

We find three significant results of policy relevance. First, adoption decisions made across the two periods are significantly and positively correlated. Second, significant complementarities (synergies) and substitutions (tradeoffs) exist among the SAI practices, indicating the importance of promoting these technologies as packages. Third, we observe that covariates that drive adoption differ significantly among practices and appear to reflect synergistic and tradeoff interrelationships among the practices across time. Particularly, farm size drives the adoption of many of the SAI practices, while off-farm income has the reverse effect. These results contribute to the debate within the development economics community on designing effective extension programs because efforts to promote adoption of one practice may appear to discourage the adoption of another practice if the interdependency between the practices is overlooked. The findings are highly relevant for policy design across sub-Saharan Africa, including Ethiopia, given economic growth in that region is intertwined with smallholder agricultural productivity amidst increasing land degradation and climate change.

## 2. Farmers’ adoption decision

Most technology adoption studies in developing countries apply expected utility theory [8, 17, 24, 36–38], predicting that households choose a technology or a package of techniques that will maximize their expected utility [39]. We assume that smallholder farmers maximize their expected benefit from adoption compared to non-adoption of SAI practices. The expected benefits could include labour or input saving and increases in output because of improved land productivity or reduced soil erosion [17, 40].

## 3. The intertemporal multivariate probit model

Adoption decisions of SAI practices are interdependent and intertemporal. Smallholder farmers deal with various agricultural production constraints, necessitating the adoption of both input-intensive and NRM practices. A few recent studies have employed a static multivariate probit model to reveal such interrelationships [8, 9, 25]. However, technology adoption decisions and their driving factors are inherently dynamic. Here, ‘dynamic’ refers to the intertemporal effects of adoption across the two periods. Therefore, we apply an intertemporal multivariate probit (MVP) model that accounts for correlation in unobserved and unmeasured factors (error terms in the expected utility functions) among practices and across time.

The intertemporal MVP model consists of eight binary choice equations in each period (*t* = 1, 2), giving 16 binary choices. The eight binary choices represent the SAI practices: chemical fertilizer, improved seed, organic manure, SWC measures, minimum tillage, crop residues, legume rotation, and legume intercropping. Following [41], the general model can be written as:

$$y{*}_{itk}=\beta {'}_{tk}x_{itk}+\varepsilon_{itk},\;t=1,2;k=1,\ldots 8$$ (1)

$$y_{itk}=1\;\mathrm{if}\;y{*}_{itk}>0\;\mathrm{and}\;0\;\mathrm{otherwise},$$ (2)

$$\varepsilon_{i}=[\varepsilon_{it1}\ldots \varepsilon_{itk}]∼MVN(0,R),$$ (3)

$$R=[\begin{array}{cccc} 1 & \rho_{t12} & \cdots & \rho_{t1K}\\ \rho_{t12} & 1 & & \vdots \\ \vdots & & \ddots & \\ \rho_{t1K} & \cdots & & 1 \end{array}]$$ (4)

where: y*_itk_ is a latent variable that captures the expected benefit to farm household *i* from adopting a particular SAI practice *k* in the period *t*. The latent variable y*_itk_ is assumed to be a linear combination of various covariates *x*_*itk*_ and the unobserved error term *ε*_*itk*_, which is denoted as *ε*_*i*_ with a 16×1 vector. The term *β*_*tk*_ is a vector of parameters to be estimated for each SAI practice *k* in period *t*. Because y*_itk_ is implicit (latent) or unobservable, the estimation is based on the observed binary choices *y*_*itk*_, which indicates whether or not a farm household *i* implemented a particular SAI practice *k* in period *t*.

The error terms *ε*_*i*_ jointly follow a multivariate normal (MVN) distribution each with mean zero and a variance-covariance matrix *R*. The modelling approach uses a 16×16 matrix *R*, with variances on the leading diagonal normalized to one and the off-diagonal values symmetrical. The off-diagonal error matrices describe the correlation of unobserved factors relating to the interdependencies of the SAI practices over the two periods. A positive correlation indicates a latent factor reflecting complementary technologies and a positive temporal effect of technology, whereas a negative correlation indicates a latent factor reflecting substitution and a negative temporal effect of technology. The maximum-likelihood function of the multivariate normal distribution involves a 16-dimensional integration and is thus done by simulation methods [41].

To ensure the robustness of our results, we also estimated the MVP model in Eq (1) by controlling lagged effects of SAI practice adoption in subsequent period. The MVP model with lagged adoption effects has the following form:

$$y{*}_{it_{2}k}=\delta {'}_{t_{1}k}y_{it_{1}k}+\beta {'}_{t_{2}k}x_{it_{2}k}+\varepsilon_{it_{1,2}k},\;i=1,2\ldots n;k=1,\ldots 8$$ (5)

where: $y{*}_{it_{2}k}$ is a latent variable that captures the expected benefit to farm household *i* from adopting a particular SAI practice *k* in the second period t_2_. The term $y_{it_{1}k}$ captures the lagged SAI practice adoption in the initial period t_1_. The term *δ*’ is a vector of parameters to be estimated for the lagged adoption effects in the initial period t_1_ and *β*’ is the vector of parameters to be estimated for the explanatory variables in the second period t_2_. Other terms are as defined earlier.

## 4. Data and adoption covariates

### 4.1 Data

This article is based on a comprehensive farm household data collected from maize growing areas of Ethiopia. The data were collected in 2010 and 2013 by the Ethiopian Institute of Agricultural Research (EIAR) in collaboration with the International Maize and Wheat Improvement Centre (CIMMYT) using a multi-stage stratified random sampling technique that appropriately accounted for the representativeness of areas with varying maize potential such as highland, midland and lowland across four regional states of the country. Four regions were covered: Amhara, Oromia, Benishangul-Gumuz and the Southern Nations, Nationalities and Peoples (SNNP). In the first stage, study districts were purposely selected from each of the four regions based on the maize production potential and the agro-ecological suitability. In the second stage, the sample kebeles were selected using the probability proportional to size sampling technique. Finally, in the selected kebele, the probability proportional to size sampling was also used to identify total sample households. After cleaning and balancing of the panel data, we used a balanced panel of 2031 farm households from 179 villages (kebeles) across 37 districts (*woredas*) in 2009/2010 and 2012/2013 production seasons. The surveys used to collect these data were comprehensive and included detailed information about production activities, technology adoption covariates, sustainable agricultural intensification practices, and farm management practices.

The data on sustainable farming practices and farm-related covariates were collected at the plot level. Typically, farm households vary the size and type of plot they allocate to maize production over different periods which makes it difficult to analyze plot-level variables in a panel structure. Thus, we collapsed the plot-level variables into a farm (household-level) and analyzed the adoption of the practices at the farm (household) level. Such a household-level analysis is common and institutive in empirical research with panel data [42–45].

### 4.2 Technology adoption covariates

A range of socioeconomic, farm characteristics, institutional and personal factors can drive the adoption of SAI practices (Table 1). We include several such covariates in our intertemporal adoption model following the existing theoretical and empirical literature [2, 7, 8, 21, 37, 40, 46–48]. A few studies provide an estimate of the prior direction of influence of these covariates on SAI practices [8, 40]. We briefly describe these adoption covariates in the context of our study.

**Table 1:** Variable lists and descriptive statistics.

Variable name	Variable definition	2009/2010		2012/2013	
		Mean	SD	Mean	SD
**Sustainable agricultural intensification (SAI) practices**					
*Input-intensive practices*					
Chemical fertilizer	= 1 if farmer applied chemical fertilizer, 0 otherwise	0.66	0.47	0.73	0.45
Improved seeds	= 1 if maize seeds used are improved varieties, 0 otherwise	0.34	0.47	0.66	0.47
*Natural resource management (NRM) practices*					
Manure	= 1 if farmer used animal manure, 0 otherwise	0.51	0.50	0.48	0.50
SWC	= 1 if farmer used soil and water conservation (SWC) practices, 0 otherwise	0.23	0.42	0.32	0.47
Crop residues retention	= 1 if farmer left any crop residues on farm in previous season, 0 otherwise	0.29	0.46	0.18	0.39
Minimum tillage	= 1 if farmer’s average ploughing frequency was at least two, 0 if above two	0.16	0.36	0.13	0.33
Legume rotation	= 1 if farmer rotated maize with legumes, 0 otherwise	0.12	0.32	0.07	0.26
Legume intercropping	= 1 if farmer intercropped maize with legumes, 0 otherwise	0.16	0.36	0.28	0.45
*Total number of SAI practices*	*Total number of SAI practices used per farm household*	*2*.*46*	*1*.*21*	*2*.*84*	*1*.*30*
**Drivers of SAI practices**					
Family labour	Household labour availability converted into adult equivalent	4.92	1.96	5.08	1.91
Family education	Average education level at the household (years of schooling) for age > = 7	2.99	2.01	3.06	1.95
Education level of head	Education level of the household head (years of schooling)	3.00	3.31	2.98	3.32
Farm size	Farm size allocated for maize in hectares	0.91	0.82	0.74	0.67
Lack of oxen	= 1 if farmer has no oxen, 0 otherwise	0.19	0.39	0.26	0.44
TLU	Total livestock holding in tropical livestock units	6.28	5.80	6.00	5.51
Off-farm income	Share of off-farm cash in total cash revenue	0.22	0.28	0.25	0.29
Age	Age of the household head	42.05	12.71	44.63	12.79
Access to institutions	Number of institutions farmer is member of in the village	2.64	1.52	2.67	2.03
Slope of the field	Type of slope as perceived by farmer (1 = flat, 2 = medium, 3 = steep)	1.36	0.49	1.35	0.52
Altitude	Altitude on which the household is located in meters above sea level (100 meter)	17.73	2.67	17.73	2.67
Tenure	Share of owned land area allocated in total maize area	0.82	0.33	0.89	0.26

*Notes*: SD is Standard deviation.

Socio-economic factors can affect technology adoption. We include the age and education of the farming household head. We also control for the education level of other household members to capture the intra-household dynamics in the adoption decision [46, 49, 50]. Farm size (maize area), total livestock units (TLU), and adult equivalent labour are included as indicators of resource availability. We include policy and institutional-related variables such as off-farm income, tenure status and access to supportive institutions for the household.

Farm characteristics can influence the management practices they implement to increase yields [51–53]. Thus, we include altitude to capture agro-ecological/climate differences between farming households across the country. We also control the slope of the farm as a covariate of adoption.

### 4.3 Technology adoption patterns over time

Table 1 presents descriptive statistics for SAI practices adoption over time. The descriptive statistics show that input-intensive technologies are adopted more than the NRM practices over the two periods (Table 1; S1 Fig). About 66% of the sample farmers used chemical fertilizer for the 2009/2010 production season and 73% for the 2012/2013 production season. The adoption of improved maize seeds increased from 34% in 2009/2010 production season to 66% in 2012/2013 production season. Among the NRM practices, the adoption of SWC increased from 23% in the first season to 32% in the second season. Likewise, the adoption of maize-legume intercropping increased from 16% in the first season to 28% in the second season. Farmers sustained the adoption of manure over time, but it is often applied around the homestead. The least adopted practice by the sample farmers is the maize-legume rotation with 5% dis-adoption over time. Farmers also dis-adopted crop residue retention and minimum tillage over time.

## 5. Results and discussion

### 5.1 Synergies and tradeoffs between practices

Before presenting the drivers of SAI practices adoption, we discuss the results of the error term correlation matrix, which provides information on the unobserved (latent) factors driving the interrelationships between the SAI practices. Based on the likelihood ratio test (*χ*^2^ (120) = 1778.47; P < 0.0001), we reject the null hypothesis of zero or no correlation between the error terms at a 1% significance level. This result suggests that the intertemporal MVP model is preferred over the single-equation probit models. In this section, we focus on the latent factor that is driving tradeoffs and synergies from the simultaneous adoption of the SAI practices as well as the intertemporal effects in conditioning the sequential adoption of the practices. A positive and negative correlations indicate latent complementary and substitution between SAI practices, respectively. The positive and negative latent correlation across time could be interpreted as a sequential technology adoption decision. The latent positive correlation across time could relate to the positive effect of experience and learning of technology that may drive the future use of the same or different technology. In contrast, the latent negative correlation across time could be related to a negative experience from using the technology leading to dis-adoption.

Table 2 presents the results of interrelationships and intertemporal adoption of the SAI practices based on the latent (error) correlation matrix from the intertemporal MVP model. The estimation results reveal evidence of significant interdependencies among the practices. The top left-hand panel gives the latent correlation between SAI practices when *t* = 1. The lower right-hand panel gives the latent correlation between practices when *t* = 2. The lower left panel offers the latent correlation between SAI practices, *across time*. Hence, the leading diagonal of the lower-left panel shows the correlation for each SAI practice with itself across the two periods. Our results demonstrate that the latent factor that is driving the probability of adoption of each SAI practice is significantly correlated across time. These are shown by the positive and highly statistically significant latent correlation of each practice over time as shown in the leading diagonal of the lower-left hand panel of Table 2 (e.g. $CF_{t_{2}}$ vs. $CF_{t_{1}}$, $CR_{t_{2}}$ vs. $CR_{t_{1}}$, etc.). We also confirm this latent correlation result by controlling actual lagged adoption effects of each SAI practice in the adoption decision of the subsequent period (S1 Table). The results demonstrate that adoption of each practice in the first panel period had a statistically significant positive effect on the adoption of the practice in the second panel period. The results underscore the positive effects of learning over time in conditioning the adoption behavior of farmers through good experience and learning about the SAI practices.

**Table 2:** Correlation matrix for the intertemporal multivariate probit model for adoption of SAI practices.

		2009/2010 = *t*_1_								2012/2013 = *t*_2_							
		$CF_{t_{1}}$	$IS_{t_{1}}$	$M_{t_{1}}$	$SWC_{t_{1}}$	$CR_{t_{1}}$	$MT_{t_{1}}$	$LR_{t_{1}}$	$LI_{t_{1}}$	$CF_{t_{2}}$	$IS_{t_{2}}$	$M_{t_{2}}$	$SWC_{t_{2}}$	$CR_{t_{2}}$	$MT_{t_{2}}$	$LR_{t_{2}}$	$LI_{t_{2}}$
**2009/2010 = t**_**1**_	$CF_{t_{1}}$	1															
	$IS_{t_{1}}$	0.367*** (0.036)	1														
	$M_{t_{1}}$	-0.127*** (0.037)	-0.158*** (0.036)	1													
	$SWC_{t_{1}}$	0.001 (0.041)	-0.023 (0.041)	0.126*** (0.039)	1												
	$CR_{t_{1}}$	-0.023 (0.039)	-0.238*** (0.038)	0.060 (0.038)	0.004 (0.042)	1											
	$MT_{t_{1}}$	-0.389*** (0.042)	-0.385*** (0.045)	0.090** (0.045)	0.133** (0.049)	0.140*** (0.046)	1										
	$LR_{t_{1}}$	0.171*** (0.051)	0.202*** (0.046)	-0.079* (0.047)	-0.010 (0.052)	0.009 (0.050)	-0.050 (0.059)	1									
	$LI_{t_{1}}$	0.105** (0.045)	-0.333*** (0.042)	0.057 (0.043)	0.110** (0.046)	0.064 (0.045)	0.164*** (0.050)	0.071 (0.053)	1								
**2012/2013 = t**_**2**_	$CF_{t_{2}}$	0.636*** (0.028)	0.137*** (0.042)	-0.017 (0.039)	0.053 (0.043)	-0.017 (0.041)	-0.198*** (0.046)	0.004 (0.052)	0.257*** (0.047)	1							
	$IS_{t_{2}}$	0.43*** (0.034)	0.249*** (0.039)	-0.045 (0.038)	-0.045 (0.041)	-0.086** (0.040)	-0.296*** (0.044)	0.133** (0.050)	0.103** (0.046)	0.689*** (0.026)	1						
	$M_{t_{2}}$	-0.036 (0.037)	0.013 (0.037)	0.183*** (0.035)	0.072* (0.039)	-0.048 (0.038)	-0.035 (0.044)	-0.131*** (0.046)	-0.046 (0.042)	-0.069* (0.038)	0.018 (0.037)	1					
	$SWC_{t_{2}}$	0.118*** (0.040)	-0.019 (0.039)	0.064* (0.038)	0.179*** (0.040)	0.053 (0.040)	0.013 (0.047)	-0.002 (0.048)	0.100** (0.044)	0.231*** (0.040)	0.149*** (0.039)	0.067* (0.037)	1				
	$CR_{t_{2}}$	-0.077* (0.044)	-0.148*** (0.045)	0.005 (0.042)	0.042 (0.046)	0.180*** (0.042)	0.063 (0.050)	-0.021 (0.053)	-0.025 (0.050)	-0.002 (0.045)	-0.087** (0.043)	-0.103** (0.041)	0.059 (0.042)	1			
	$MT_{t_{2}}$	-0.283*** (0.048)	-0.307*** (0.052)	0.032 (0.049)	0.087 (0.053)	0.096* (0.050)	0.571*** (0.041)	0.009 (0.063)	0.113** (0.054)	-0.346*** (0.046)	-0.387*** (0.045)	-0.079 (0.048)	0.036 (0.051)	-0.046 (0.056)	1		
	$LR_{t_{2}}$	0.115** (0.059)	0.131** (0.055)	-0.078 (0.054)	-0.028 (0.061)	-0.011 (0.056)	-0.032 (0.069)	0.286*** (0.060)	0.021 (0.064)	-0.007 (0.059)	0.013 (0.058)	-0.085 (0.054)	-0.079 (0.057)	-0.060 (0.062)	-0.097 (0.075)	1	
	$LI_{t_{2}}$	0.175*** (0.039)	-0.168*** (0.040)	0.095** (0.038)	0.129*** (0.041)	0.030 (0.041)	-0.018 (0.047)	-0.040 (0.049)	0.388*** (0.039)	0.257*** (0.039)	0.093** (0.040)	0.116*** (0.037)	-0.012 (0.039)	0.164*** (0.042)	0.020 (0.050)	-0.027 (0.057)	1

*Notes*: CF = Chemical fertilizer, IS = Improved seed, M = Manure, SWC = Soil and water conservation, CR = Crop residues, MT = Minimum tillage, LR = Legume rotation, LI = Legume intercropping. Subscript *t*_1_ = 2009/2010 and *t*_2_ = 2012/2013.

*, ** and *** are significant at 10%, 5% and 1% level. Standard errors are in parenthesis.

The results reveal important synergies and tradeoffs from the simultaneous adoption of input-intensive technologies and NRM practices. For example, for 2009/2010, chemical fertilizer is found to have a statistically significant negative association with manure and minimum tillage, but a statistically significant positive association with improved seed, legume rotation and legume intercropping (top left panel of Table 2). For 2012/2013, chemical fertilizer is found to have a statistically significant negative association with manure and minimum tillage, but a statistically significant positive association with improved seed, SWC, and legume intercropping (bottom right panel of Table 2). Our findings are consistent with recent studies that provide evidence on the interdependent adoption of those practices in a static framework [8, 9, 26]. Thus, we build on this empirical evidence to challenge the widely-held misperception that these two types of practices are incompatible. Indeed, smallholder farmers adopt input-intensive and NRM practices as complements (synergies) or as substitutes (tradeoffs) depending on their needs and prior experience.

We find both statistically positive and negative latent correlations among the input-intensive and NRM practices for Ethiopian farmers. There are four statistically significant possible correlations in unobservable effects among the practices (Table 3). For example, the use of improved seed is positively correlated four times with inorganic fertilizer (Table 3). Legume rotation is highly positively correlated with the use of improved seed. Minimum tillage and crop residue retention are strongly negatively correlated with the use of improved seed. Legume intercropping was found to have positive or negative correlations with improved maize seed. This result could be related to the type of maize variety (long or short type) planted in the season because farmers tend to intercrop legumes with certain types of varieties. In this study, we are unable to disaggregate the improved maize seed by the types of maize varieties grown, long or dwarf type. A disaggregated analysis of improved maize seed would offer deeper insights.

**Table 3:** Number of statistically significant intertemporal correlations among SAI practices.

	*Input-intensive practices*		*Natural resource management practices*				
	Chemical fertilizer	Improved seed	Manure	Soil and water conservation	Crop residues	Minimum tillage	Legume rotation
Improved seed	**++++**						
Manure	- -	-					
Soil and water conservation	**++**	**+**	**++++**				
Crop residues	-	- - - -	-	**ns**			
Minimum tillage	- - - -	- - - -	**+**	**+**	**++**		
Legume rotation	**++**	**+++**	- -	**ns**	**ns**	**ns**	
Legume intercropping	**++++**	**++**- -	**++**	**+++**	**+**	**++**	**ns**

*Notes*: A ‘+’ and ‘–’ are the number of statistically significantly positive (synergistic) and negative (tradeoffs) correlations, respectively. ‘ns’ is not statistically significant. The maximum possible number of correlations is four (see Table 2). These correlations are used to develop SAI package types and their determinants of adoption (see S3 Table). The practices with greater number of ‘+’ or ‘-’ indicate strong synergies and tradeoffs, respectively.

**Fig 1:**
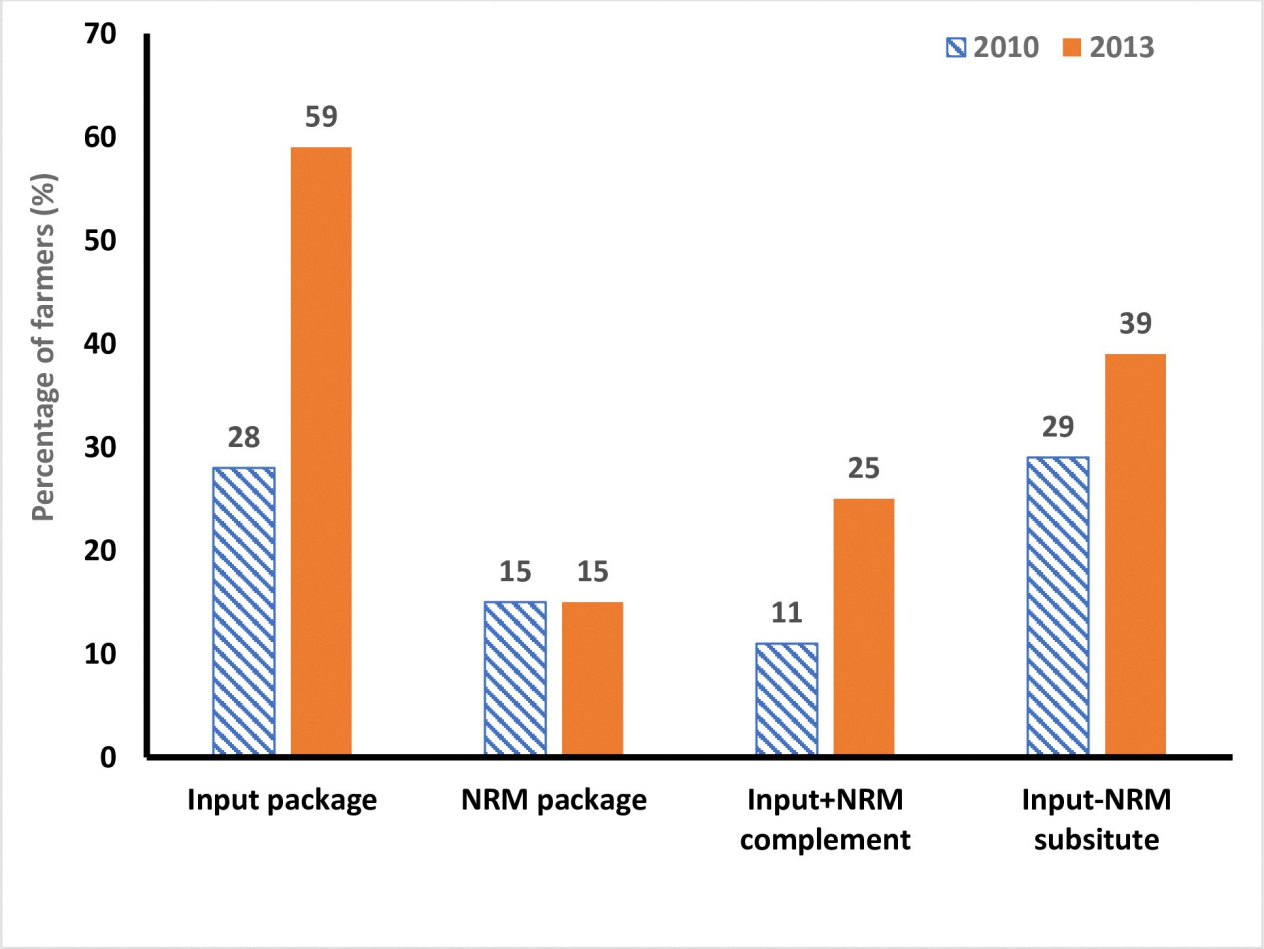
Packages of input-intensive and natural resource management (NRM) practices in Ethiopia.

Based on the MVP prediction, we identified different packages of SAI practices (Fig 1; S2 Table). A large percentage of farmers adopted the input-intensive package (inorganic fertilizer and improved seed) over the panel period. We also observed more adoption of the package in 2012/2013 production season compared to the 2009/2010 production season. The adoption of NRM package was about 15% and this has not changed over the panel period. However, the input-intensive and NRM complements (synergies) increased from 11% in 2009/2010 production season to 25% in the 2012/2013 production season. The input-intensive and NRM substitutes (tradeoffs) increased from 29% in the 2009/2010 production season to 39% in the 2012/2013 production season. Overall, these results demonstrate that beneficial synergies of substitutions or synergistic complementarities of technology packages can be exploited using input-intensive practices (improved seed or chemical fertilizer) and NRM practices [1, 2, 5, 7, 25]. Therefore, policy makers and development agents should promote such synergetic and tradeoff technology bundles/packages in the specific niches of farmers’ agro-ecological and farming systems. It is imperative to assess whether the observed interdependencies in the practices are related to the drivers of adoption. We discuss these issues in the next sections.

### 5.2 Drivers of intertemporal technology adoption

In this section, we focus on the driving factors of adoption with particular attention to possible differences in the covariates between input-intensive and NRM practices. We also investigate whether the drivers (covariates) reflect the implicit patterns of complementarity and tradeoffs among the practices across time.

Table 4 presents the coefficient estimates from the intertemporal MVP model. The coefficient estimates indicate the direction and significance of the association between the practices and covariates for adoption and the parameter estimates should not be interpreted as structurally causal. We also ensure the robustness of our covariate results by estimating a lagged MVP probit model (S1 Table).

**Table 4:** Coefficient estimates of the intertemporal multivariate probit model.

Drivers of SAI practices adoption	2009/2010				2012/2013			
	Chemical fertilizer	Improved seeds	Manure	SWC	Chemical fertilizer	Improved seeds	Manure	SWC
Family labour	0.032* (0.017)	0.062*** (0.017)	-0.019 (0.017)	-0.020 (0.019)	0.008 (0.017)	0.078*** (0.017)	0.032** (0.017)	0.011 (0.017)
Family education	0.069*** (0.019)	0.088*** (0.019)	-0.017 (0.018)	0.024 (0.020)	0.054** (0.019)	0.025 (0.019)	-0.005 (0.018)	0.022 (0.019)
Education level of head	-0.002 (0.012)	-0.018 (0.012)	0.001 (0.012)	-0.030** (0.013)	-0.012 (0.012)	-0.002 (0.012)	-0.020* (0.011)	0.007 (0.012)
Farm size	0.232*** (0.043)	0.137*** (0.038)	0.022 (0.038)	-0.082 (0.045)	0.375*** (0.052)	0.379*** (0.052)	0.082* (0.046)	0.112** (0.048)
Lack of oxen	-0.177** (0.076)	0.031 (0.082)	-0.273*** (0.080)	-0.029 (0.089)	-0.283*** (0.071)	-0.250*** (0.073)	-0.132* (0.072)	0.046 (0.076)
TLU	-0.013** (0.006)	-0.005 (0.006)	0.0003 (0.006)	0.0001 (0.007)	-0.011 (0.007)	-0.014** (0.007)	-0.002 (0.006)	0.014** (0.006)
Off-farm cash	-0.406*** (0.099)	-0.241** (0.110)	-0.392*** (0.105)	-0.329** (0.121)	-0.277** (0.101)	-0.344*** (0.104)	-0.146 (0.102)	-0.380*** (0.112)
Age	-0.011*** (0.003)	-0.012*** (0.003)	0.007** (0.003)	-0.007** (0.003)	-0.014*** (0.003)	-0.016*** (0.003)	0.0003 (0.003)	-0.009*** (0.003)
Access to institutions	0.084*** (0.019)	0.078*** (0.019)	-0.021 (0.019)	0.109*** (0.021)	0.065*** (0.016)	0.049*** (0.016)	0.026* (0.015)	0.055*** (0.015)
Slope of the field	0.026 (0.057)	0.006 (0.060)	-0.103* (0.059)	0.353*** (0.063)	0.093 (0.056)	-0.119** (0.056)	-0.117** (0.056)	0.356*** (0.057)
Altitude	0.047*** (0.011)	-0.045*** (0.012)	0.037*** (0.011)	0.008 (0.012)	0.059*** (0.011)	0.010 (0.011)	0.0003 (0.011)	0.029** (0.012)
Tenure	-0.078 (0.082)	0.013 (0.089)	-0.034 (0.087)	0.093 (0.099)	-0.124 (0.113)	-0.267** (0.115)	0.201* (0.112)	0.206* (0.120)
Constant	-0.478* (0.249)	0.069 (0.260)	-0.459* (0.246)	-1.203*** (0.273)	-0.177 (0.265)	0.747** (0.264)	-0.232 (0.258)	-1.629*** (0.276)
**Drivers of SAI practices adoption**	**2009/2010**				**2012/2013**			
	**Crop residues**	**Minimum tillage**	**Legume rotation**	**Legume intercropping**	**Crop residues**	**Minimum tillage**	**Legume rotation**	**Legume intercropping**
Family labour	-0.010 (0.018)	-0.067*** (0.021)	0.057** (0.022)	0.022 (0.020)	0.006 (0.019)	-0.041* (0.022)	0.032 (0.025)	0.059*** (0.017)
Family education	-0.035* (0.020)	-0.021 (0.022)	0.022 (0.024)	-0.002 (0.022)	0.025 (0.022)	-0.007 (0.025)	0.007 (0.028)	-0.034* (0.020)
Education level of head	0.030** (0.012)	0.042*** (0.014)	0.010 (0.015)	0.015 (0.014)	0.018 (0.013)	0.035** (0.015)	0.016 (0.017)	0.007 (0.012)
Farm size	0.178*** (0.039)	0.066 (0.042)	0.195*** (0.044)	0.095** (0.044)	0.078 (0.052)	-0.172*** (0.060)	0.209*** (0.060)	0.039 (0.050)
Lack of oxen	-0.121 (0.086)	0.296*** (0.091)	0.136 (0.104)	0.243** (0.089)	0.030 (0.085)	0.390*** (0.088)	0.073 (0.108)	0.078 (0.075)
TLU	-0.001 (0.006)	-0.013 (0.008)	0.004 (0.007)	-0.029*** (0.009)	0.015** (0.007)	0.003 (0.008)	-0.001 (0.009)	-0.021*** (0.007)
Off-farm cash	-0.144 (0.114)	0.431*** (0.123)	-0.067 (0.144)	-0.185 (0.124)	-0.036 (0.123)	-0.009 (0.133)	0.111 (0.158)	0.057 (0.105)
Age	0.004 (0.003)	0.015*** (0.003)	-0.009** (0.004)	-0.001 (0.003)	-0.007** (0.003)	0.011*** (0.003)	-0.006 (0.004)	0.0003 (0.003)
Access to institutions	-0.116*** (0.021)	-0.055** (0.024)	0.050** (0.024)	0.016 (0.022)	-0.014 (0.017)	-0.043** (0.021)	0.006 (0.021)	0.006 (0.015)
Slope of the field	0.214*** (0.061)	0.225*** (0.071)	-0.406*** (0.088)	0.039 (0.069)	0.283*** (0.062)	0.126* (0.070)	0.001 (0.086)	0.096* (0.057)
Altitude	-0.016 (0.011)	-0.156*** (0.014)	-0.030** (0.015)	0.019 (0.014)	0.021 (0.013)	-0.198*** (0.016)	-0.040** (0.017)	0.023* (0.012)
Tenure	0.274*** (0.096)	0.113 (0.107)	0.265** (0.126)	0.188* (0.110)	-0.095 (0.127)	0.039 (0.147)	0.109 (0.171)	0.238** (0.119)
Constant	-0.707** (0.260)	0.885*** (0.309)	-0.725** (0.333)	-1.645*** (0.309)	-1.564*** (0.298)	1.754*** (0.356)	-1.069** (0.397)	-1.530*** (0.283)

*Notes*: *, ** and *** are significant at 10%, 5% and 1% probability level. N = 2031; log likelihood = -15539.56; Wald *χ*^2^ (192) = 1301.49***; likelihood ratio test of rho *χ*^2^ (120) = 1778.47***. To reduce simulation bias, the number of simulation draws (50) was set above the square root of the number observations [41]. Standard errors are in parenthesis.

These results indicate that the interrelationships among SAI practices, their complementary and tradeoff patterns, as well as temporal effects, play roles in conditioning the drivers of the adoption process. The results underline the relevance of understanding both the intertemporal (sequential adoption process) and interrelationships (simultaneous adoption process) among practices when investigating the drivers of technology adoption. Likewise, the derivers of adoption decision in the second period (2012/2013) are highly consistent (statistically significant coefficients and sign) with the lagged MVP model results (S1 Table).

Socio-economic, farm, institutional and personal characteristics drive SAI practices over time. Family labour availability has a positive association with many of the SAI practices but a negative association with minimum tillage. The results reinforce the critical role of labour in driving the adoption of SAI practices [2, 6, 7, 46]. Conversely, the adoption of minimum tillage appears to be a response to a lack of labour and saving labour is one of the core principles of conservation agriculture [40, 54].

Education is also a key part of the story. A higher level of education of family members has a significant positive association with the probability of adoption of inputs, such as improved seed or chemical fertilizer, and their NRM substitute like crop residue retention. The education level of the household head is positively associated with the adoption of reduced (minimum) tillage and crop residue retention but negatively associated with SWC and organic manure. These results underscore the fact that characteristics of the family is important in making adoption decisions, contrary to the common, skewed, emphases given to the household head. The results are consistent with [50], who revealed the education of family members to be more critical than the education level of the household head for fertilizer adoption in Ethiopia. The education level of family members was found to have a positive association with the adoption of SWC measures not only in sub-Saharan Africa but also in the Honduran hillsides [17]. Thus, understanding the education level of household members sheds deeper insights on the adoption of SAI practices.

The age of the household head is negatively associated with adoption of SAI practices except for minimum tillage. Given the rapid youth migration to urban areas for better wages, enhancing the sustainability of land degradation in Ethiopia could be undermined especially for the labour-intensive NRM practices.

Farm size is found to drive the adoption of many SAI practices while off-farm income is found to have the reverse effect. The exception is minimum tillage in which farm size has a negative association while off-farm income has a positive association. It should be noted that minimum tillage is a labour-saving conservation agricultural practice. The result suggests that off-farm work might take away farm labour that could have been used to implement SAI practices. Alternatively, off-farm income may not have been invested in the implementation of SAI practices. These results point to the need to be cautious when promoting linkages between farm and non-farm activities in developing countries where markets are imperfect [55, 56]. Lack of oxen or livestock is negatively associated with the adoption of modern inputs (chemical fertilizer and improved seed) and their NRM complement, organic manure. Farmers appear to implement minimum tillage and legume intercropping when they face oxen constraints.

Institutions (formal and informal) are also key drivers of SAI practices adoption. Having higher institutional capital as proxied by the number of supportive institutions has a significant positive association with the adoption of SWC measures, improved seed, chemical fertilizer, and legume rotation. However, institutional capital is negatively associated with the adoption of crop residue retention and minimum tillage, which are the two pillars of conservation agriculture. This result could be due to lack of mechanized farm power such as two-wheel tractor to implement conservation agriculture practices [57–59]. Ethiopian farmers often use inefficient traditional oxen-drawn power for ploughing their farm. Farmers are also more likely to use NRM practices such as crop residues, organic manure, legume intercropping and legume rotation when they own their farms relative to rented farms. This result signifies the role of tenure security for ensuring sustainable agriculture. However, farmers tend to use improved seed on rented farms.

Farm characteristics also significantly influence the adoption behavior of farmers. Consistent with the previous literature [8, 17, 23, 47], the probability of adoption of SWC, minimum tillage and crop residue increases in steeper farms where they are most needed to curb soil erosion. However, improved seed, legume rotation, and organic manure are less likely to be implemented in steeper farms. Finally, chemical fertilizer and organic manure are more likely applied in the high land areas, whereas improved seeds, minimum tillage and legume rotation are more likely implemented in the low land areas. These results could be related to the niche of the agro-ecology of the farming system that is more favorable to certain SAI practices than others, which could partly reflect exposure to varying climate or weather shock [60]. Such niche-targeting of SAI practices were also observed for Kenyan maize farmers [8].

Overall, we find factors that drive adoption differ among practices across time and such patterns appear to reflect the complementarity and trade-offs implicit in those practices. We also find consistent results for the various SAI packages and their determinants (S1 and S3 Tables). These empirical insights are important for designing effective extension programs because efforts to promote adoption of one practice may appear to discourage the adoption of another practice if the interdependency among the practices across time is overlooked. Overall, the drivers of adoption from the intertemporal MVP model (Table 4; S1 Table) and the panel probit models of the SAI packages (S3 Table) are highly consistent and could inform food security policy in Africa.

## 6. Conclusions and policy implications

We used a comprehensive two-wave panel data of smallholder maize producers in Ethiopia to analyze synergies, tradeoffs, and drivers in the adoption of sustainable farming practices. Two types of SAI practices were considered: input-intensive and natural resource management (NRM) practices. The input-intensive practices include improved maize seed and chemical fertilizer, and the NRM practices include soil and water conservation (SWC) measures, organic manure, crop residue retention, reduced tillage, legume rotation and legume intercropping. We applied an intertemporal MVP model that allows both correlations between practices and intertemporal correlation of the practices adopted reflecting simultaneous and sequential adoption, respectively.

We reveal three significant findings of relevance to policy. First, we find a significant positive latent correlation between each practice across time. The result underscores the positive temporal effect (lagged adoption effects) in conditioning the continued adoption of the practices. This result is new in the context of sustainable agricultural intensification process. While previous research on duration analysis captured intertemporal adoption decisions, the intertemporal correlation among technologies that could reflect beneficial synergistic effects were overlooked. These findings point to the need for continuity of programs aimed at promoting SAI practices for widespread adoption by providing persistent incentives and extension services to smallholder farmers.

Second, we find clear evidence of complementarities and tradeoffs among SAI practices. Thus, we build on emerging evidence [8, 9, 61] to challenge the widely-held misperception that these two types of SAI practices are incompatible. Indeed, smallholder farmers adopt modern inputs and NRM practices as complements (synergies) or as substitutes (tradeoffs) depending upon their current needs and prior experience of farming. These results underline the importance of implementing both input-intensive and NRM practices as compatible technology packages. Promoting these SAI packages as predicted by the MVP model could bring a feasible solution to low agricultural productivity in predominantly capital-deficient and environmentally-degraded production systems of sub-Saharan Africa.

Third, we find clear evidence that factors that drive adoption differ between practices and across time. We also observe that the factors that drive SAI practices appear to reflect the interrelationships in those practices across time. For instance, we find farm size increases the likelihood of adoption of several SAI practices while off-farm income has the reverse effect. These contrasting effects of farm and off-farm activities in the adoption process caution when designing policy programs to promote SAI practices.

Overall, our findings contribute to the debate within the development economics community in designing effective programs. This is because efforts to promote adoption of one practice may appear to discourage adoption of another practice due to lack of understanding of the underlying interdependencies among the practices across time. Understanding the drivers of adoption of SAI practices would also be critical. The results offer insights for public and private sectors that support the widespread adoption of these practices by smallholder farmers. For example, Ethiopia’s current initiative of cluster farming may help increase farm size by consolidating fragmented farms [62, 63] to encourage the adoption of multiple SAI practices. The findings are highly relevant for African policymakers since the continent’s economic growth is essentially intertwined with smallholder agricultural productivity amidst increasing soil degradation. As also argued in the empirical literature [64–67], the underutilization of external inputs such as chemical fertilizer in the African smallholder sector calls for a balanced use of a portfolio of SAI practices, which include both input-intensive and NRM practices to ensure a sustainable productivity growth. A ‘piecemeal’ linear approach of promoting either subset of these technologies may not bring about intended sustainable agricultural intensification outcomes: increasing productivity while ensuring environmental sustainability.

More research on the tangible impacts of the intensive use of these practices, including the combinations that may lead to a maximal benefit to smallholder farmers (e.g. reduce production risks and food insecurity) would be useful in guiding niche-tailored program design [68]. Understanding the spatial adoption decisions of the practices would also shed further insight [3]. Modelling interrelationships of SAI practices with long panel periods would also offer more insights [69]. Further research should investigate these issues in depth.

## Supporting information

S1 Fig Adoption of input-intensive and natural resource management (NRM) practices in Ethiopia. (TIF)

S1 Table Coefficient estimates of the multivariate probit model estimates with lagged SAI practice adoption. (DOCX)

S2 Table Variable lists and descriptive statistics for individual and SAI package types. (DOCX)

S3 Table Coefficient estimates of probit model estimates for SAI packages. (DOCX)

S1 Data (XLSX)
