# Supplementary figures and images for: Dynamics of multiple sustainable agricultural intensification practices adoption: Application of the intertemporal multivariate probit model
Source: PLoS One. 2025 Feb 7;20(2):e0314172. doi: 10.1371/journal.pone.0314172 (PMC11805428; doi:10.1371/journal.pone.0314172)

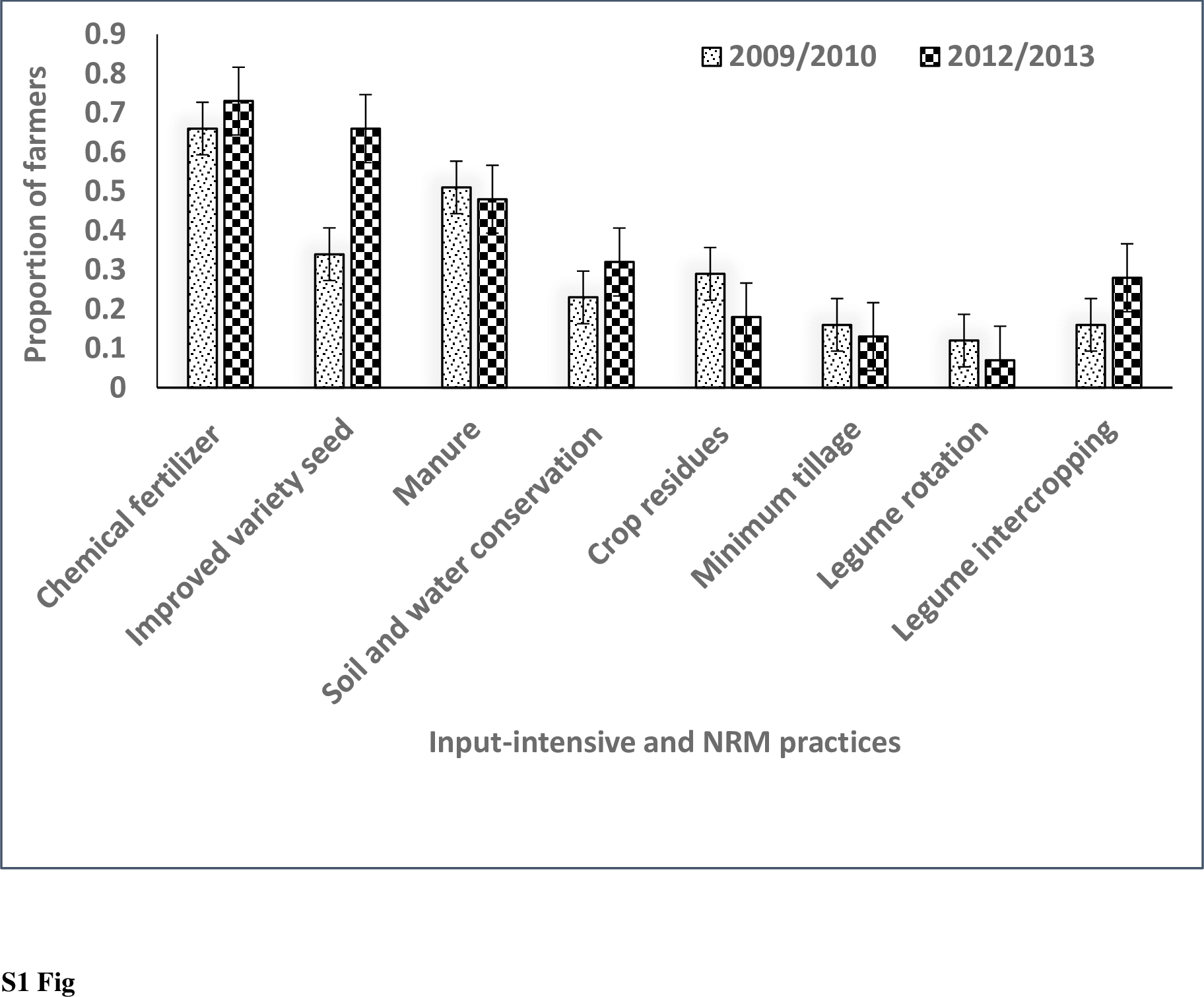

Supplement: S1 Fig — (TIF) [file pone.0314172.s001.tif]
