# Supplementary material for: Dynamics of multiple sustainable agricultural intensification practices adoption: Application of the intertemporal multivariate probit model
Source: PLoS One. 2025 Feb 7;20(2):e0314172. doi: 10.1371/journal.pone.0314172 (PMC11805428; doi:10.1371/journal.pone.0314172)
Supplement: S1 Table — (DOCX) [file pone.0314172.s002.docx]

**S1 Table. Coefficient estimates of the multivariate probit model estimates with lagged SAI practice adoption**

| **Drivers of SAI practices adoption** | **SAI practices adoption in 2012/2013 production season** | | | | | | | |
| --- | --- | --- | --- | --- | --- | --- | --- | --- |
|  | **Chemical fertilizer** | **Improved**  **maize seed** | **Manure** | **SWC** | **Crop residues** | **Minimum tillage** | **Legume rotation** | **Legume intercropping** |
| *Lagged practice adoption* | 0.908***  (0.067) | 0.422***  (0.064) | 0.296***  (0.057) | 0.325***  (0.069) | 0.297***  (0.070) | 1.033***  (0.093) | 0.536***  (0.110) | 0.713***  (0.079) |
| Family labour | -0.001  (0.020) | 0.071***  (0.018) | 0.034**  (0.017) | 0.012  (0.018) | 0.008  (0.020) | -0.022  (0.025) | 0.028  (0.025) | 0.057***  (0.018) |
| Family education | 0.050**  (0.022) | 0.015  (0.020) | -0.006  (0.018) | 0.023  (0.019) | 0.028  (0.022) | 0.0005  (0.027) | 0.004  (0.028) | -0.033  (0.020) |
| Education level of head | -0.019  (0.014) | 0.000  (0.012) | -0.020*  (0.011) | 0.008  (0.012) | 0.017  (0.013) | 0.030*  (0.017) | 0.014  (0.017) | 0.003  (0.012) |
| Farm size | 0.382***  (0.060) | 0.387***  (0.054) | 0.084*  (0.046) | 0.115**  (0.049) | 0.056  (0.052) | -0.234***  (0.067) | 0.200***  (0.061) | 0.015  (0.051) |
| Lack of oxen | -0.295***  (0.084) | -0.294***  (0.076) | -0.126*  (0.073) | 0.035  (0.077) | 0.042  (0.085) | 0.398***  (0.098) | 0.077  (0.110) | 0.029  (0.078) |
| TLU | -0.012  (0.008) | -0.018**  (0.007) | -0.003  (0.006) | 0.014**  (0.006) | 0.015**  (0.007) | 0.007  (0.009) | -0.002  (0.009) | -0.019**  (0.007) |
| Off-farm cash | -0.308**  (0.118) | -0.350***  (0.109) | -0.120  (0.104) | -0.383***  (0.113) | -0.037  (0.124) | -0.123  (0.149) | 0.120  (0.161) | 0.043  (0.110) |
| Age | -0.015***  (0.003) | -0.015***  (0.003) | 0.0001  (0.003) | -0.009***  (0.003) | -0.007**  (0.003) | 0.008**  (0.004) | -0.006  (0.004) | 0.0001  (0.003) |
| Access to institutions | 0.069***  (0.018) | 0.066***  (0.016) | 0.031**  (0.014) | 0.055***  (0.015) | -0.019  (0.017) | -0.072***  (0.023) | 0.010  (0.021) | 0.009  (0.015) |
| Slope of the field | 0.116*  (0.066) | -0.111*  (0.059) | -0.131**  (0.056) | 0.371***  (0.058) | 0.288***  (0.062) | 0.132*  (0.079) | 0.003  (0.087) | 0.124**  (0.059) |
| Altitude | 0.054***  (0.012) | 0.018  (0.011) | -0.003  (0.011) | 0.026**  (0.012) | 0.024*  (0.013) | -0.172***  (0.018) | -0.035**  (0.017) | 0.018  (0.012) |
| Tenure | -0.142  (0.135) | -0.323**  (0.123) | 0.201*  (0.114) | 0.206*  (0.121) | -0.096  (0.129) | 0.076  (0.165) | 0.098  (0.174) | 0.240*  (0.126) |
| Constant | -0.521*  (0.296) | 0.523*  (0.275) | -0.317  (0.260) | -1.700***  (0.278) | -1.692***  (0.304) | 1.128***  (0.394) | -1.213***  (0.400) | -1.567***  (0.293) |

*Notes*: *, ** and *** are significant at 10%, 5% and 1% probability level. N=2031; log likelihood = -7523.86; Wald $\chi^{2}$ (104) = 1203.36***; likelihood ratio test of rho $\chi^{2}$ (28) =412.27***. To reduce simulation bias, the number of simulation draws (50) was set above the square root of the number observations [41]. Lagged practice adoption refers to the dynamics of past adoption decisions of each SAI practice in the previous panel period (2009/2010) production season.
