# Supplementary material for: Dynamics of multiple sustainable agricultural intensification practices adoption: Application of the intertemporal multivariate probit model
Source: PLoS One. 2025 Feb 7;20(2):e0314172. doi: 10.1371/journal.pone.0314172 (PMC11805428; doi:10.1371/journal.pone.0314172)
Supplement: S2 Table — (DOCX) [file pone.0314172.s003.docx]

**S2 Table. Variable lists and descriptive statistics for individual and SAI package types**

| **Major SAI packages predicted by the MVP model** | **Package description** | **2009/2010** | | **2012/2013** | |
| --- | --- | --- | --- | --- | --- |
|  |  | **Mean** | **SD** | **Mean** | **SD** |
|  | | | | | |
| Input-intensive | =1 if farmer used intensive inputs (chemical fertilizer and improved maize varieties), 0 otherwise | 0.28 | 0.45 | 0.59 | 0.49 |
| NRM practices | =1 if farmer used at least three of the NRM practices (manure, SWC, crop residues retention, minimum tillage, legume rotation & legume intercropping), 0 otherwise | 0.15 | 0.36 | 0.15 | 0.36 |
| Input-intensive +NRM complements (synergies) | = 1 if farmer used intensive inputs (maize seed & chemical fertilizer) and any of the NRM complements (SWC and legume rotation), 0 otherwise | 0.11 | 0.32 | 0.25 | 0.43 |
| Input-intensive-NRM substitutes (tradeoffs) | = 1 if farmer used intensive inputs (maize seed & chemical fertilizer) and any of the NRM substitutes (crop residues, manure & minimum tillage), 0 otherwise | 0.29 | 0.45 | 0.39 | 0.49 |

*Notes*: SD is Standard deviation. The SAI packages are identified from complementary and substitution effects of the MVP model predictions (see Table 2 and Table 3).
