# Supplementary material for: Dynamics of multiple sustainable agricultural intensification practices adoption: Application of the intertemporal multivariate probit model
Source: PLoS One. 2025 Feb 7;20(2):e0314172. doi: 10.1371/journal.pone.0314172 (PMC11805428; doi:10.1371/journal.pone.0314172)
Supplement: S3 Table — (DOCX) [file pone.0314172.s004.docx]

**S3 Table. Coefficient estimates of probit model estimates for SAI packages.**

| **Drivers of SAI packages** | **Input-intensive** |  | **NRM** |  | **Input-intensive +NRM complement** |  | **Input-intensive-NRM substitute** |  |
| --- | --- | --- | --- | --- | --- | --- | --- | --- |
|  | Coefficient | Std. error | Coefficient | Std. error | Coefficient | Std. error | Coefficient | Std. error |
| Family labour | 0.067*** | 0.013 | 0.037** | 0.017 | 0.044*** | 0.014 | 0.036** | 0.014 |
| Family education | 0.058*** | 0.014 | -0.006 | 0.019 | 0.038** | 0.016 | 0.037** | 0.015 |
| Education level of head | 0.0004 | 0.009 | 0.016 | 0.012 | 0.012 | 0.010 | 0.003 | 0.009 |
| Farm size | 0.150*** | 0.031 | 0.104** | 0.040 | 0.141*** | 0.033 | 0.265*** | 0.034 |
| Lack of oxen | -0.081 | 0.057 | 0.147** | 0.075 | 0.042 | 0.065 | -0.110* | 0.062 |
| TLU | -0.009* | 0.005 | -0.013** | 0.006 | 0.001 | 0.005 | -0.004 | 0.005 |
| Off-farm cash | -0.332*** | 0.079 | -0.436*** | 0.110 | -0.321*** | 0.095 | -0.404*** | 0.087 |
| Age | -0.010*** | 0.002 | -0.002 | 0.003 | -0.010*** | 0.002 | -0.005** | 0.002 |
| Access to institutions | 0.079*** | 0.012 | 0.009 | 0.016 | 0.091*** | 0.013 | 0.006 | 0.013 |
| Slope of the field | 0.020 | 0.043 | 0.305*** | 0.055 | 0.154*** | 0.048 | 0.085* | 0.046 |
| Altitude | 0.004 | 0.008 | -0.028** | 0.011 | -0.002 | 0.010 | -0.013 | 0.009 |
| Tenure | -0.035 | 0.072 | 0.262** | 0.103 | 0.117 | 0.086 | 0.061 | 0.078 |
| Constant | -0.530** | 0.191 | -1.450*** | 0.265 | -1.496*** | 0.223 | -0.590** | 0.208 |
| Log likelihood | -2661.09 |  | -1664.01 |  | -1845.99 |  | -2501.47 |  |
| Wald $\chi^{2}$(12) | 206.95*** |  | 77.07*** |  | 150.70*** |  | 159.09*** |  |

*Notes*: *, ** and *** are significant at 10%, 5% and 1% probability level. N=4062.
